# Emotion Regulation Questionnaire for Cross-Gender Measurement Invariance in Chinese University Students

**DOI:** 10.3389/fpsyg.2020.569438

**Published:** 2020-11-04

**Authors:** Ying Zhang, Yufang Bian

**Affiliations:** ^1^Collaborative Innovation Center of Assessment toward Basic Education Quality, Beijing Normal University, Beijing, China; ^2^Institute of Mental Health and Education, Beijing Normal University, Beijing, China; ^3^Child and Family Education Research Center, Beijing Normal University, Beijing, China

**Keywords:** emotion regulation, cognitive reappraisal, expression suppression, across gender, measurement invariance

## Abstract

**Objectives:**

Emotion regulation has been extensively studied in various areas of psychology. The Emotion Regulation Questionnaire (ERQ) was developed to assess two specific constructs associated with emotion control—cognitive reappraisal and expression suppression (Gross and John, 2003). The instrument displayed sound psychometric properties; however, to date, inquiry regarding the measure’s characteristics has been limited. This study aims to measure cross-gender invariance [measurement invariance (MI)] in Chinese undergraduates using the ERQ.

**Methods:**

This study measured the psychometric properties of the ERQ in a sample of 847 Mainland China undergraduates (401 males and 446 females) through confirmatory factor analysis. The tests of MI were used to examine potential structural differences based on gender.

**Results:**

The findings supported the measure’s original structure with all demographic groups and demonstrated exceptional fit. Additional normative data for gender and ethnic groups are included as well. The results also supported the use of the instrument in future research.

**Conclusion:**

The two-factor structure in the ERQ establishes a cross-gender equivalence between males and females in Chinese college students. This study supports the use of the instrument in future research.

## Introduction

Emotion regulation implies the process that individuals use to regulate, experience, and express their emotions (Gross, 2002; John and Gross, 2007; Wang et al., 2020). Using emotion regulation strategies, individuals could alter their emotions in physiological activities, subjective experiences, and behavior (Ochsner and Gross, 2008; Miao, 2009; Gratz et al., 2015). Individuals regulate their emotions using the emotion regulation strategy, which enables them to improve, maintain, or reduce one or several emotional reactions (Gross, 1998; Dunsmore et al., 2013). Emotion regulation can influence individuals’ physical health (e.g., sleep quality) (Minkel et al., 2012), mental health (e.g., social anxiety and other negative emotions) (Goldin et al., 2012), interpersonal relationships (e.g., partnership and parent–child relationship) (English et al., 2012; Shi et al., 2019). Reportedly, individual emotion regulation could appear and often play a role in daily life and various interpersonal interactions (Gross et al., 2006). Emotion regulation has become a pressing issue in the field of psychology.

Successful emotion regulation strategies are crucial for an individual’s emotion (Cai et al., 2012), social support (English et al., 2012; Goldin et al., 2012), and subjective well-being (Parkinson and Totterdell, 1999; Gross and John, 2003; McRae et al., 2012). To clearly and directly assess emotion regulation strategies, Gross (1998) developed the Emotion Regulation Questionnaire (ERQ) based on the process model of emotion regulation [i.e. ERQ, compiled by Gross (1998) at Stanford University, United States, which focuses on the frequency of individual utilization of emotion regulation strategies by measuring two dimensions: “cognitive reappraisal” and “expression suppression,” ^1^ (Chinese version)]. Cognitive reappraisal is an antecedent-focused strategy and often tries to reinterpret events positively (e.g., *When I’m faced with a stressful situation, I make myself think about it in a way that helps me stay calm*) (John and Gross, 2004). Expressive suppression, however, attempts to suppress, hide, or reduce emotional expression (e.g., *I keep my emotions to myself*) (John and Gross, 2004). Gross’s ERQ comprises 10 items, including 6 items for measuring the cognitive reappraisal dimension and 4 measuring the expression suppression dimension. In recent years, ERQ has been extensively used in the measurement of special and normal groups and has been translated into different languages and widely used worldwide (Liu et al., 2017; Lotfi et al., 2019; Pastor et al., 2019; Wang et al., 2020). ERQ is acceptable to excellent levels of internal consistency reliability across various types of participants (posttraumatic stress disorder, anxiety disorders, normal adolescents, and young adults) (Gross and John, 2003; Wiltink et al., 2011; Spaapen et al., 2014; Preece et al., 2019).

The effects of cognitive reappraisal and expressive suppression are manifold depending on the cultural background. In the Western cultural background, the impact of cognitive reappraisal is more positive such as better social support and lower level of psychopathology symptoms (Moore et al., 2008; Joormann and Gotlib, 2010; McRae et al., 2012), whereas the impact of expressive suppression is more negative such as higher level of depression and anxiety (Moore et al., 2008; Eftekhari et al., 2009). However, in the Asian cultural background, cognitive reappraisal could be an ineffective strategy for some minority groups experiencing oppression, and expressive suppression appears to be less harmful (Soto et al., 2012; Su et al., 2015; Wang et al., 2020). Indeed, most studies that investigated the ERQ’s psychometric properties are under Western cultural background (Australian Bureau of Statistics, 2017), and a few have focused on the Asian cultural background (e.g., Mainland China) (Preece et al., 2019). Wang et al. (2007) explored the ERQ’s psychometric properties in Chinese college students, and Wang et al. (2020) tested the ERQ’s psychometric properties in Chinese rural-to-urban migrant adolescents and young adults; both studies found that the reliability and validity of ERQ fulfilled the requirements of psychometrics.

The research testing measurement invariance (MI) across different populations using the confirmatory factor analysis (CFA) has highlighted the significance of identifying discrepancies in factor and parameter characteristics and assessing how this could affect and distort between-group comparisons (Meredith, 1993). Wang et al. (2007) and Wang et al. (2020) focused on Oriental culture under the background of people’s emotion regulation strategies, and their studies’ impact on the measurement tool laid the foundation. Although both studies mentioned above in China reported worthwhile findings, the consideration of MI did not receive attention. Thus, it is crucial to determine whether the underlying traits measured by the measurement (e.g., ERQ in this study) are equivalent across different groups. For example, the ERQ measuring emotion regulation could exhibit variance across gender. Despite this inconsistency, measurement has always been a combination of males and females without distinction, and the latent construct of emotion regulation being measured could be observed in the male group but not in the female group, or *vice versa*. In this instance, variance is expected, and perhaps, the construct cannot be measured in the female or male group. Consequently, the scale could be an excellent measure of the latent construct of emotion regulation in a male population; however, the mean score comparisons between the male and female groups are relatively worthless because of measurement non-equivalence across the items. Such issues are of key significance in cross-gender research and when examining potential intergroup differences (e.g., based on gender, ethnicity, or age) in psychological constructs measured through self-reporting (Little, 1997; Gregorich, 2006). In addition, comparisons of gender differences based on the ERQ or studies of the impact of emotional regulation strategy between different genders should be based on the measurement equivalence of the scale. When the study was based on the scale to conduct further research and found differences between different genders, one should first consider from the angle of exploring ERQ measurement equivalence between different gender groups, that is, the scale to participants of different genders was measured on the equivalence, only to make the equivalence scale further valuable. However, to date, no equivalence study based on this scale has been reported among different genders in Chinese cultural background, and this study is conducted on such considerations.

## This Study

This study uses tests of model invariance to determine whether the scale illustrates consistent measurement characteristics across two specific demographic comparisons—male and female undergraduate participants. The normative data for these gender groups in an undergraduate sample are included to provide further information about how the questionnaire performs across varying participant groups. It is hypothesized that this study will support the two-subscale structure illustrated in a previous research, and the measure will demonstrate invariance across gender comparison groups.

## Materials and Methods

### Participants and Procedure

We enrolled junior and senior students from a university in Beijing. A total of 882 participants (47.01% males), aged 19–23 years, were enrolled [mean (*M*_*age*_) = 21.31, standard deviation (*SD*) = 1.09]. The sample encompassed 93.42% of individuals who reported their ethnicity as Han, and a further 6.58% classified themselves as belonging to an ethnic minority. To control ordering effects, the order of questionnaire administration was counterbalanced in each study. All participants were given information outlining the purpose and possible drawbacks of participation before completing the measures, as well as the opportunity to decline participation if they desired. Participants completed all measures and returned the questionnaires to research assistants before leaving the classroom.

### Measures

In this study, the ERQ comprised 10 items. It includes two dimensions—cognitive reappraisal factor (six items; items 1, 3, 5, 7, 8, and 10) and expression suppression factor (four items; items 2, 4, 6, and 9). The ERQ is primarily used to evaluate individual emotion regulation strategies. We used the Likert seven-point scoring method for the items. The higher the score, the higher the frequency of using emotion regulation strategy. The internal consistency (Cronbach’s *α*) in this study was 0.825.

### Statistical Analysis

#### Missing Data

The original sample included 882 Chinese college students; however, as 35 failed to respond to all ERQ items, they were excluded from the analysis. A total of 847 valid questionnaires (401 males and 446 females) were collected (effective rate: 96.03%).

#### Analytic Stages

Our analyses contained the following two stages: (i) CFA tested the fit of the emotional regulation model; and (ii) MIs of the emotional regulation model were assessed, from the CFA, across gender.

#### Stage 1: Model Evaluation in CFA

CFA was conducted for the Emotional Regulation model, and the CFA was specified and estimated using Mplus 8.0 software (Muthén and Muthén, 1998-2017). Based on previous studies, we used some fit indices to assess the overall fit of the models; these included chi-square (*χ*^2^), comparative fit index (CFI), Tucker–Lewis index (TLI), root mean square error of approximation (RMSEA), and standardized root mean square residual (SRMR). The values >0.90 for the CFI and TLI and <0.08 for the RMSEA and SRMR indicated an adequate fit (Kline, 2010).

#### Stage 2: Model Specification

Following the generally accepted practice, we assessed the fit of each model by examining multiple fit indices (Kline, 2010). When examining factorial invariance, we followed the established procedures (Meredith, 1993; Gregorich, 2006; Meredith and Teresi, 2006), which were used in the related literature (Engdahl et al., 2011; Wang et al., 2013a). If configural invariance (baseline model, Model A) is supported, further restrictive constraints could be imposed on the model, as was performed in the conventional multiple group CFA invariance test. First, factor loadings were constrained to be equal across gender to test metric or weak invariance (Model B). In addition, a *χ*^2^ difference test was conducted to assess if the baseline model was significantly different from the constrained model. A non-significant *χ*^2^ difference test indicated that factor loadings were invariant across gender, thereby satisfying metric invariance. Furthermore, based on the metric invariance model, intercepts were constrained to be equal across gender to build Model C, a test of scalar or strong invariance. Model D included the restrictions from Model C plus the additional constraint of equal item error variances across the two genders (invariant error variance or strict invariance). Subsequent to Model D, residual error variances were not constrained to be equal across timepoints (Grouzet et al., 2006). Thus, Model E was compared with Model C to preserve nested model testing. Model E comprised the constraints from Model C plus the additional constraint of equal factor variances across the two genders (invariant factor variances). During testing, except for the baseline model (Model A), the first two invariance testing analyses were also called MI, while the next invariance testing analyses were called structural invariance.

### Data Analysis

Statistical analyses were performed using SPSS 19.0, JASP-0.11.1.0 (^2^; Marsman and Wagenmakers, 2017; Wagenmakers et al., 2017a; Wagenmakers et al., 2017a,b), and Mplus 8.0 (Muthén and Muthén, 1998-2017). JASP-0.11.1.0 software was primarily used to analyze the kurtosis and skewness of items. Using Mplus 8.0 software, we used the CFA of the ERQ, compared the fitting index, and obtained the best factor model to fit the Chinese college students. In addition, significant skewness and kurtosis values were obtained for each item (*p* < 0.01). We selected the robust maximum-likelihood estimation method for unbiased estimation of non-normal distribution data for data analysis (Satorra and Bentler, 2001). The robust ML estimator with a mean-adjusted *χ*^2^ (maximum likelihood parameter estimates with standard errors and a mean-adjusted *χ*^2^ test statistic) was selected, as these provide parameter estimates that are robust to non-normality (Satorra and Bentler, 2001; Wang et al., 2013a). Furthermore, we use the corrected scaled *χ*^2^ difference test to compare the nested models (Satorra and Bentler, 2001).

We evaluated the fit of each model by examining multiple fit indices (Kline, 2010; Wang et al., 2012). We used the Satorra Bentler chi-square statistic (S-B*χ*^2^), RMSEA, SRMR, TLI, and CFI. On the basis of extensive simulation studies conducted by Hu and Bentler (1999), it appears that good-fitting models have CFI and TLI values greater than 0.95, RMSEA values less than 0.06, and less than 0.08 (Wang et al., 2012). The corrected scaled chi-square difference test developed by Satorra and Bentler (2001); Muthén and Muthén (1998-2017) was used to compare nested models. However, tests of the change in CFI (i.e., ΔCFI) are superior to chi-square (Δ*χ*^2^) difference tests of invariance because they are not affected by the sample size (Cheung and Rensvold, 2002; Meade et al., 2008). Thus, the corrected scaled chi-square difference test and change in CFI were used to compare nested models. When both results contradict each other, however, we primarily depended on results of CFI differences.

According to the suggestion of Cheung and Rensvold (2002), the change in CFI was chosen to evaluate the measurement invariance. When ΔCFI < 0.01, it implies that the invariance hypothesis cannot be rejected, and the model fits well; when 0.01 ≤ ΔCFI ≤ 0.02, it implies that the degree of the model has a moderate deterioration, which cannot reveal that the difference exists and is significant; when ΔCFI ≥ 0.02, it signifies a significant difference (Cheung and Rensvold, 2002; Meade et al., 2008; Wang et al., 2013b), and the standard of the nested model is ΔCFI < 0.01, ΔTLI < 0.01 (Wang et al., 2012, Wang et al., 2013b).

### Ethics Statement

In this study, the core variables were participants’ ERQ scores, and we collected the data in the classroom. Written informed consent was obtained from all principals and participants in this study. The protocol and questionnaires used were approved by the university’s Institutional Review Board.

## Results

### Descriptive Statistics

Table 1 lists the average scores measured by the ERQ and standardized factor loads for each item. Significant multivariate skewness and kurtosis were found (*p* < 0.05, based on univariate and multivariate tests). In the ERQ, the real score was 20–53 (male: 36.83 ± 6.118; female: 32.98 ± 5.732), and the male score was significantly higher than the female score (*t* = 3.054, *p* < 0.01, *d* = 0.46). In the cognitive reappraisal factor score, the male score was 16.02 ± 2.659, while the female score was 14.95 ± 2.802; thus, the male and female scores revealed no statistically significant difference (*t* = 1.223, *p* = 0.171). In the expression suppression factor score, the male score was 22.01 ± 3.754, while the female score was 18.65 ± 4.002; the male score was significantly higher than that of the females (*t* = 3.124, *p* < 0.01, *d* = 0.42). In this study, Cronbach *α* was 0.825 in the ERQ, and the coefficient *α* of cognitive reappraisal and expression suppression was 0.831 and 0.778, respectively.

**TABLE 1:** Descriptive statistics results of Emotion Regulation Questionnaire (ERQ).

**Item**		***M (SD)***	**Skewness**	**Kurtosis**	**Factor load**		***t***	***p***	***Cohen’s d***
					**CR**	**ES**			
*Cognitive reappraisal*									
Item 1		3.76 (1.779)	−0.22	0.55	0.591**				
Item3		3.87 (1.754)	−0.21	0.61	0.698**				
Item 5		3.72 (1.782)	−0.25	0.37	0.657**				
Item 7		3.81 (1.791)	−0.25	0.39	0.589**				
Item 8		3.75 (1.802)	−0.19	0.48	0.592**				
Item 10		3.69 (1.793)	−0.13	0.61	0.563**				
*Expression suppression*									
Item 2		4.21 (1.901)	−0.41	0.89		0.631**			
Item 4		4.16 (1.330)	−0.53	0.87		0.602**			
Item 6		3.91 (1.324)	−0.47	0.89		0.603**			
Item 9		3.87 (1.135)	−0.55	0.91		0.594**			
*Scores for different gender*									
*Total scores*	*Males*	36.83 (6.118)					3.054**	<0.01	0.46
	*Females*	32.98 (5.732)							
*CR scores*	*Males*	16.02 (2.659)					1.223	0.171	—
	*Females*	14.95 (2.802)							
*ES scores*	*Males*	22.01 (3.754)					3.124**	<0.01	0.42
	*Females*	18.65 (4.002)							

**p < 0.01. CR, cognitive reappraisal; ES, expression suppression.

Item analysis was used to discriminate each item (Table 2). (i) A critical ratio (decision values of the high- and low-score groups) was used and the correlation of the total items to test the discrimination of each item. We defined the first 27% of the score in the ERQ as the high-score group, while the latter 27% as the low-score group. (ii) Each item score difference in the high- and low-score groups was compared in this study. The results revealed that the ERQ scores in the high- and low-score groups were statistically significant, and the correlation of the total items were 0.38–0.62 (*p* < 0.01).

**TABLE 2:** The *t*-test of high- and low-score group for each item and total item correlation of ERQ.

**Item**	***t***	***p***	**Total item correlation**
*Cognitive reappraisal*			
Item 1	9.46	<0.001	0.530^∗∗^
Item 3	12.77	<0.001	0.582^∗∗^
Item 5	10.54	<0.001	0.577^∗∗^
Item 7	11.39	<0.001	0.589^∗∗^
Item 8	12.32	<0.001	0.621^∗∗^
Item 10	11.55	<0.001	0.502^∗∗^
*Expression suppression*			
Item 2	10.71	<0.001	0.501^∗∗^
Item 4	8.92	<0.001	0.384^∗∗^
Item 6	11.23	<0.001	0.522^∗∗^
Item 9	10.05	<0.001	0.598^∗∗^

*^∗∗^*p* < 0.01.*

### Stage 1: Confirmatory Factor Analysis

**FIGURE 1:**
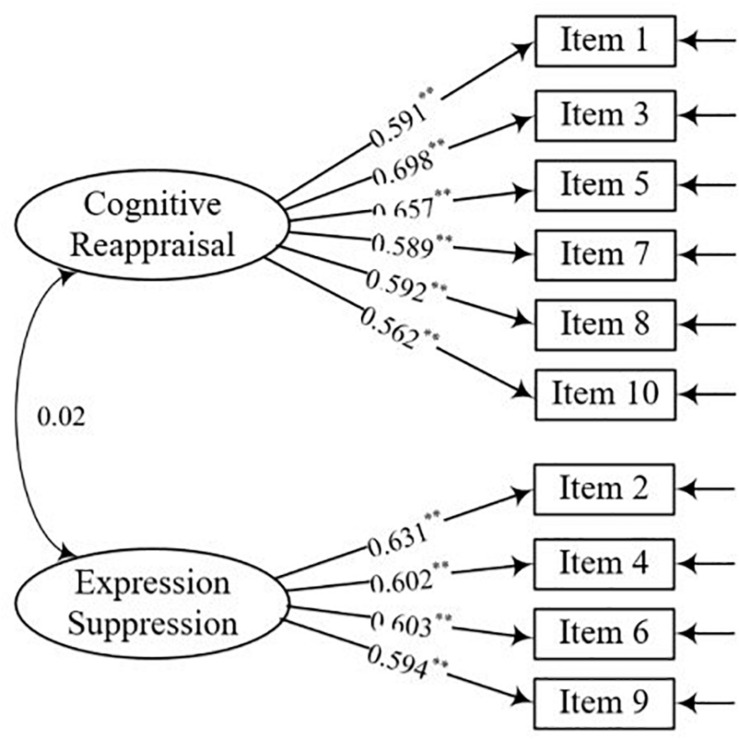
Standardized factor loadings for total sample confirmatory factor analysis (CFA).

The CFA results (Figure 1) revealed that S-B *χ*^2^/*df* = 5.95, *p* = 0.004, CFI = 0.93, TLI = 0.92, RMSEA = 0.056, and SRMR = 0.038. Specifically, for males, the CFA results revealed that S-B *χ*^2^/*df* = 3.49, *p* = 0.002, CFI = 0.94, TLI = 0.93, RMSEA = 0.043, and SRMR = 0.051. For females, the CFA results revealed that S-B *χ*^2^/*df* = 3.66, *p* = 0.002, CFI = 0.95, TLI = 0.93, RMSEA = 0.059, and SRMR = 0.044 (Table 3).

**TABLE 3:** Two-factor structure model fitting results in ERQ.

	**S-Bχ^2^/*df***	**CFI**	**TLI**	**RMSEA**	**SRMR**
Total	5.95	0.934	0.929	0.056	0.038
Male	3.49	0.941	0.932	0.043	0.051
Female	3.66	0.945	0.934	0.059	0.044

*ERQ, emotion regulation questionnaire.S-Bχ^2^, Satorra–Bentler scaled χ^2^; *df*, degrees of freedom; TLI, Tucker–Lewis index; CFI, comparative fit index; RMSEA, root-mean-square error of approximation; SRMR, standardized root mean squared residual.*

### Stage 2: Measurement Invariance Testing Across Gender

The results from the MI across gender revealed that all five steps of MI testing resulted in significant *χ*^2^ (*ps* < 0.01), excellent (CFIs > 0.95, TLIs > 0.090), and equivalent fit indices (ΔCFIs < 0.01, ΔTLIs < 0.01). Moreover, all goodness-of-fit indices suggested that all models assuming different degrees of invariance were acceptable (Table 4).

**TABLE 4:** Goodness-of-fit indices of the compared models.

**MI model**	**S-Bχ^2^**	***df***	**RMSEA [90%CI]**	**CFI**	**TLI**	**Model comparison**	**ΔCFI**	**ΔTLI**
Model-A	443.589	68	0.051 [0.046, 0.058]	0.964	0.946		—	—
Model-B	457.902	76	0.049 [0.044, 0.056]	0.964	0.949	B vs. A	0	0.003
Model-C	488.271	84	0.050 [0.044, 0.057]	0.961	0.948	C vs. B	−0.003	−0.001
Model-D	513.678	94	0.045 [0.042, 0.055]	0.957	0.951	D vs. C	−0.004	0.002
Model-E	567.237	97	0.043 [0.040, 0.054]	0.953	0.952	E vs. D	−0.004	0.001

*Model A indicates no parameters constrained to be equal across groups; model B, factor loadings constrained to be equal; model C, observed variable intercepts and factor loadings constrained to be equal; model D, residual variances, factor loadings, and observed variable intercepts constrained to be equal; model E, factor variances and covariances, factor loadings, and observed variable intercepts constrained to be equal. CI indicates confidence interval. *df*, degrees of freedom; TLI, Tucker–Lewis index; CFI, comparative fit index; RMSEA, root-mean-square error of approximation; SRMR, standardized root mean squared residual; S-Bχ^2^, Satorra–Bentler scaled χ^2^.*

#### Configural Invariance (Model A)

In the configural MI testing, the factor load and the intercept of observation variables were performed for free estimation. In this study, each fitting index of Model A fulfilled the measurement standard (CFI ≥ 0.90; TLI ≥ 0.90), thereby establishing the configural invariance, and Model A fulfilled the requirements as the next MI analysis baseline model (Table 4).

#### Metric Invariance (Model B)

After passing the configural invariance testing, the factor load MI was set according to Model A, and both groups of corresponding factor loads were constrained to be equal to test the weak invariance model. After increasing the factor load equal constrain, if the data fitting situation did not reach the standard in statistics, the constrain was not removed. In this study, comparing the CFIs and TLIs of Model B and Model A, the |ΔCFI| and |ΔTLI| values were 0 and 0.003. As shown in Table 4, the model fitted well, and the MI test continued.

#### Scalar Invariance (Model C)

Based on the construction of Model B, we set the measurement intercepts of two groups equally (Model C). As shown in Table 4, we compared the CFIs and TLIs of Model C and Model B, the |ΔCFI| and |ΔTLI| values were 0.003 and 0.001, and the model fitted well, thereby the MI test continued.

#### Residual Error Invariance (Model D)

Based on Model C, we constrained residual error variances across the groups. Then, we compared CFIs and TLIs values of Model D and Model C, the |ΔCFI| and |ΔTLI| values were 0.004 and 0.002. As shown in Table 3, the model fitted well, thereby the MI test continued.

#### Invariant Factor Variances (Model E)

The final test of this study was to test structural invariance (Model E), which additionally constrained factor variances and covariances (not residual variances), tested against Model C. As shown in Table 4, |ΔCFI| and |ΔTLI| values of the two models mentioned above were 0.004 and 0.001, respectively, implying that the factor variance MI was established.

## Discussion

This study first tested the two-factor structure of the emotion regulation using the CFA among Mainland China college students. The item analysis revealed that the distinction and discrimination of the items were acceptable, which is consistent with previous studies that used the CFA to compare alternative structures of emotion regulation among Chinese rural-to-urban migrant youth (Wang et al., 2020). The Cronbach’s *α* of ERQ total scores and subscales was acceptable (0.778–0.831), suggesting that the ERQ is a reliable measure of emotion regulation. The CFA results supported the two-factor structure of the ERQ, which demonstrated a clear replication with the results of most previous studies (Wang et al., 2007; Matsumoto et al., 2008). The total internal consistency *α* coefficient of the ERQ was 0.825, and each dimension was 0.831 (cognitive reappraisal) and 0.778 (expressive suppression), which is acceptable. In addition, *α* coefficients of the ERQ were similar to that in previous studies in Chinese literature (cognitive reappraisal, *α* = 0.85; expressive suppression, *α* = 0.77) (Wang et al., 2007); however, *α* coefficients of the ERQ were marginally lower than that of the rural-to-urban migrant adolescents and young adults in China (the total internal consistency *α* coefficient of the ERQ was 0.82, and each dimension was 0.82 (cognitive reappraisal) and 0.73 (expressive suppression) (Wang et al., 2020); this could be attributable to different characteristics of different groups of people.

This study examined MI across gender and compared the gender difference of emotion regulation strategy based on the ERQ. The findings demonstrated that all models assuming different degrees of invariance were acceptable, suggesting that the ERQ factors have the same meaning across gender, suggesting that comparisons across gender based on the ERQ are meaningful. This study’s results of MI across gender corroborated previous research, in which MI was found in a sample of American undergraduates (Melka et al., 2011). Furthermore, the results of this research extend the study area from the perspective of MI in Mainland China with Oriental cultural background.

Comparison of differences in ERQ scores and the two factors between males and females revealed that males’ overall emotion regulation is markedly higher than females’. Regarding cognitive reappraisal factors, no significant difference was observed between males and females, whereas, a significant difference was observed between males and females in terms of expression suppression, suggesting that males exhibit more utilization of expression suppression strategies for emotion regulation than females. Notably, previous studies have compared the emotion regulation strategy of people from various backgrounds (Sala et al., 2012). However, as related to gender, if the MI does not hold across groups, differences in observed scores may not be directly comparable. This finding is consistent with previous studies on the differences in emotion regulation between males and females (Hess et al., 2000; Parkins, 2012; Chaplin and Aldao, 2013), and, thus, our results provide additional empirical support from Mainland China for their conclusion.

Our findings provide crucial meaning for practice. First, influenced by Chinese traditional culture, undergraduates in Mainland China are not good at expressing their emotions, which remind college administrators to be concerned about undergraduates, teach them emotion regulation strategies and interpersonal communication strategies, and provide them with opportunities to interact and practice emotion regulation strategies in their relationships, and specific educational schedules should be developed and used for this group. Second, gender differences depicted in ERQ measurement scores reflect the real differences in the cognitive reappraisal and expression suppression between males and females, rather than caused by the variance measured by the ERQ itself (Meredith and Teresi, 2006), thereby providing a comparative psychological basis for related research. Finally, it is significant that future emotion regulation measurement and invariance measurement criteria should consider this character.

This study has some limitations. First, we used a restricted sample of college students from Mainland China; thus, the results might not be entirely generalizable for all Chinese population. Second, the sample was not considered regarding other variables and, thus, was not further explained; however, it could serve as a basis for future research. Finally, we used a more appropriate parameter estimate approach (Flora and Curran, 2004; Melka et al., 2011).

## Conclusion

This study establishes the ERQ as a structurally consistent and sound measure of cognitive reappraisal and emotional suppression across gender groups. Given the popularity of emotion regulation research in recent years, attempts to elucidate mea sures of associated constructs are vital. This study provides further evidence that the ERQ is a valuable research topic. Nonetheless, continued efforts to use the instrument in future studies are highly recommended.

## Data Availability Statement

The original contributions presented in the study are included in the article/supplementary material, further inquiries can be directed to the corresponding author.

## Ethics Statement

The studies involving human participants were reviewed and approved by the Institutional Review Committee of the Collaborative Innovation Center of Assessment toward Basic Education Quality, Beijing Normal University. Participants provided their written informed consent to participate in the study.

## Author Contributions

YZ designed and executed the study, analyzed the data, and wrote the manuscript. YB collaborated with the design of the study. All authors contributed to the article and approved the submitted version.

## Conflict of Interest

The authors declare that the research was conducted in the absence of any commercial or financial relationships that could be construed as a potential conflict of interest.

## Footnotes

1 https://spl.stanford.edu/sites/g/files/sbiybj9361/f/chinese.pdf

2 https://jasp-stats.org/
